# A High-Density Genetic Map and QTL Fine Mapping for Growth- and Sex-Related Traits in Red Swamp Crayfish (*Procambarus clarkii*)

**DOI:** 10.3389/fgene.2022.852280

**Published:** 2022-02-15

**Authors:** Xin-Fen Guo, Yu-Lin Zhou, Min Liu, Zhi Li, Li Zhou, Zhong-Wei Wang, Jian-Fang Gui

**Affiliations:** ^1^ State Key Laboratory of Freshwater Ecology and Biotechnology, Hubei Hongshan Laboratory, The Innovation Academy of Seed Design, Institute of Hydrobiology, Chinese Academy of Sciences, Wuhan, China; ^2^ College of Life Sciences, University of Chinese Academy of Sciences, Beijing, China; ^3^ Key Laboratory of Ministry of Water Resources for Ecological Impacts of Hydraulic-Projects and Restoration of Aquatic Ecosystem, Institute of Hydroecology, Ministry of Water Resources, Chinese Academy of Sciences, Wuhan, China

**Keywords:** *Procambarus clarkii*, genetic linkage map, QTL mapping, growth-related traits, sex

## Abstract

Red swamp crayfish (*Procambarus clarkii*) is a commercially important species in global aquaculture and most successfully invasive freshwater shrimp in China. In order to determine the genetic basis of growth- and sex-related traits, a high-density genetic linkage map was constructed using 2b-RAD sequencing technology in a full-sib family. The consensus map contains 4,878 SNP markers assigned to 94 linkage groups (LGs) and spanned 6,157.737 cM with an average marker interval of 1.26 cM and 96.93% genome coverage. The quantitative trait locus (QTL) mapping for growth and sex traits was performed for the first time. QTL mapping uncovers 28 QTLs for growth-related traits in nine LGs, explaining 7.9–14.4% of the phenotypic variation, and identifies some potential candidate growth-related genes such as *mih*, *lamr*, *golgb1*, *nurf301*, and *tbcd1* within the QTL intervals. A single major locus for sex determination was revealed in LG20 that explains 59.3–63.7% of the phenotypic variations. Some candidate sex-related genes, such as *vps4bl*, *ssrf*, and *acot1*, were identified in the QTL intervals and found to be differentially expressed in the muscle tissues between the females and the males. Furthermore, the identified SNPs were revealed to be female heterozygotes, suggesting that red swamp crayfish might have the female heterogametic ZZ/ZW sex determination system. The present study provides a valuable resource for marker-assisted selection and genetic improvement and for further genetic and genomic research in red swamp crayfish.

## Introduction

The red swamp crayfish (*Procambarus clarkii*) was native to the United States and Mexico and was introduced to Nanjing, China, from Japan in the 1930s (Hobbs et al., 1989; Yue et al., 2008). The crayfish has become widespread in almost all forms of freshwater such as lakes, rivers, and rice paddy fields all around China in the past few decades due to its high environmental adaptability and survivability (Shen et al., 2014). Furthermore, at present, red swamp crayfish has a great market demand because of its delicious meat in China. In addition to its food value, it is also a good crustacean model organism in the studies of viral infections and environmental stress (Wu et al., 2012; Celi et al., 2013; Chen et al., 2013; Lin et al., 2013). Growth is an important trait that determines economic benefit in aquaculture. The traditional breeding model in crayfish, namely, catching and selling large individuals but maintaining small individuals as parents in rice fields, has resulted in a decrease of growth traits remarkably. Furthermore, sexual dimorphism was observed, and it showed that the males grow faster than the females, but the sex-determining mechanism of crayfish is still unclear. So, there is an urgent need to establish a precise breeding program to improve economic traits and promote the development of crayfish industry (Gui et al., 2022). Traditional genetic breeding programs mainly rely on phenotypic trait selection, which has a long breeding cycle and low selection efficiency (Zenger et al., 2017). Fast and effective breeding methods, such as marker-assisted selection (MAS), can use genetic information to select important traits, which provides convenience for genetic selection (Raina et al., 2020).

In recent years, the genetic linkage map construction and QTL mapping have become important marker-assisted selection breeding tools for their abilities to identify molecular markers or candidate genes, which are associated with traits of growth, sex, and disease resistance (Yu et al., 2015; Peng et al., 2016). In the early years, the molecular markers including AFLP, RFLP, and SSR were mainly used for construction of genetic linkage maps (Yue, 2014). However, the development of these markers is expensive and time consuming, resulting in fewer and low-density molecular markers, limiting their ability for fine mapping and localization of QTL for important traits. At present, single-nucleotide polymorphisms (SNPs) are the most abundant genetic markers in genome, which are stable, simple, and may be directly related to the phenotype (Baird et al., 2008; Shi et al., 2014). Therefore, SNPs gradually replaced the traditional molecular markers and became the best choice in the construction of the genetic linkage map (Fu et al., 2016; Peng et al., 2016; Liu et al., 2017; Feng et al., 2018; Zhang et al., 2019; Zhou et al., 2021). The development of next-generation sequencing technologies has facilitated the development of SNPs, and various genotyping methods have been developed to screen thousands of SNP markers rapidly and cheaply (Davey et al., 2011). Restriction site-associated DNA sequencing (RAD-seq) uses specific restriction enzymes to identify short fragments of DNA near the site to reduce the complexity of target genome samples (Baird et al., 2008; Davey and Blaxter, 2010). 2b-RAD-seq, a modified RAD strategy using type IIB restriction endonucleases for genome genotyping, is a relatively simple library preparation scheme (Wang et al., 2016). Compared with the traditional simplified genome technology, the 2b-RAD-seq technique can obtain a larger number of markers with identical length and higher typing accuracy and is widely applied in high-density genetic mapping construction, such as snakehead (*Channa argus*) (Liu et al., 2020), bighead carp (*Aristichthys nobilis*) (Fu et al., 2016), and sea cucumber (*Apostichopus japonicus*) (Tian et al., 2015). Consequently, we used 2b-RAD-seq to construct the genetic linkage map of red swamp crayfish in this study.

The goal of this article was to construct a high-density SNP genetic map by 2b-RAD-seq, screen QTLs associated with growth and sex, and further identify growth- and sex-related candidate genes in red swamp crayfish. Compared with other crustacean species, red swamp crayfish is more complex with 188 chromosomes (Zhang et al., 2018), which indicates that more SNP markers should be developed for high-density map construction. The result of our study will not only reveal the locations and effects of QTLs for growth and sex fundamentally but also provide a basis for marker-assisted breeding and industry promotion in *P. clarkii* in the future.

## Materials and Methods

### Mapping Family Establishment

Wild red swamp crayfish adults were collected from Hongze Lake, Jiangsu, and Poyang Lake, Jiangxi, and were raised in the National Aquatic Biological Resource Center (NABRC). The full-sib families were established by natural mating between one male and four females in one closed culture tank. The genetic relationship among these parents was evaluated using 10 highly polymorphic microsatellite loci developed based on transcriptome data. After PCR amplification, the products were separated by electrophoresis on agarose gels stained with ethidium bromide. Then, the gels were photographed under UV light for band counting, and the bands were binary coded by 1 or 0 for their presence or absence in each genotype. Finally, the genetic distance was calculated by POPGENE software (unpublished data). When the crayfish began to release eggs, every spawning crayfish was transferred to a separate tank for hatching. One family with a high genetic distance was selected as the mapping family. After 3 months of culture, a total of 130 progenies were selected, and six growth-related traits including body weight (BW), full length (FL), body length (BL), tail length (TL), carapaces width (CW), and chelae width (CHW) were measured to compare the growth rate of crayfish; Pearson’s correlation coefficient between these traits were also calculated. The sexes of crayfish were identified based on the difference between male and female pleopods. The tail muscle tissues of the parents and 130 progenies were collected and stored in absolute ethyl alcohol for DNA extraction. Total DNA was extracted by using the Genomic DNA Extraction Kit (Promega, USA), following the manufacturer’s protocol. DNA quality and concentration were assessed by 1% agarose gel electrophoresis and a UV spectrophotometer and then stored at −80°C for library construction.

### 2b-RAD Sequencing and *De Novo* Genotyping

A total of 130 offspring and two parents were used for 2b-RAD-seq library construction by the previously published protocols (Wang et al., 2012). In brief, 200 ng genome DNA of each individual was first digested in 15 µl reaction mixture with 1U BsaXI (New England Biolabs) for 45 min at 37°C. Then, 10 µL of digestion product, 0.2 µM of specific adaptors (5 pairs of adaptors per 5 samples), 0.5 mM ATP (New England Biolabs), 200U T4 DNA ligase (New England Biolabs), 2 µl 10×T4 ligase buffer (New England Biolabs), and 5.9 µl of nuclease-free water were mixed to 20 µl for ligation at 16°C for 1 h. Next, the ligation products were amplified by PCR, and the barcodes were then introduced by specific primers. The amplified products were purified using the MinElute PCR Purification Kit (QIAGEN). Next, all constructed libraries were pooled with equal amounts of product from each library to make a final library, which was then sequenced by the Illumina Hiseq Xten sequencing platform. Raw reads were initially assigned to each individual according to their specific barcodes. Then, the reads were first trimmed to remove adapter sequences and the terminal 3 bp. Next, the reads with no enzyme restriction sites, containing long homopolymers (>10 bp), ambiguous bases (N), or low-quality sequences (quality <20), or of mitochondrial origin were removed. The remaining high-quality reads with a length of 27 bp were used for *de novo* 2b-RAD-seq genotyping.

### High-Density Linkage Map Construction

The identified SNP markers that could be segregated in crayfish parents and be genotyped in at least 80% of the offspring were used for map construction. Chi-square tests were performed to check whether the markers were consistent with the expected separation ratio of 1:1 or 1:2:1. The markers with significant segregation distortion (*p* < 0.05) were removed, and slightly distorted markers were remained for map construction. The consensus linkage map was grouped and constructed by the JoinMap 4.0 program (Ooijen and Van, 2006) with a threshold logarithm of odds (LOD) score of 9.5, and the Linkage Map View package in R was used to visualize the mapping results. The expected genetic map length was calculated using *Ge1* (Fishman et al., 2001) and *Ge2* (Chakravarti et al., 1991), and the average of these two indexes was used as the predicted total genetic map length (*Ge*).

### QTL Mapping for Growth- and Sex-Related Traits

A QTL analysis of growth- and sex-related traits was performed using MapQTL6 software (Ooijen et al., 2009). Multiple QTL models (MQMs) were used to detect all the significant associations between traits and marker loci (Jansen and Stam, 1994). The LOD scores were calculated at an interval of 1 cM, and the genome-wide (significance level) and linkage group-wide (suggestive level) LOD thresholds were estimated using a permutation test (1,000 replicates) with a confidence interval of 95%. QTL was determined to be significant if the LOD score was higher than the significance threshold estimated by permutation. The percentage of phenotypic variation explained by each QTL was obtained.

### Identification and Validation of Potential Candidate Genes

The marker sequences of each QTL region were used to blast against the genome survey (unpublished data provided by Yan-he Li from Huazhong Agricultural University) and annotated by transcriptome data of crayfish and non-redundant protein sequences (NR) from the NCBI database. qPCR was performed on growth and sex candidate genes to reveal the relationship between traits and expression levels. RNA was taken from the tail muscles of three fast-growing (FG) and three slow-growing (SG) crayfish individuals, as well as from the gonads of three male and three female individuals using the SV Total RNA Isolation Kit (Promega, USA). The quality and concentration of RNA were evaluated by 1% agarose gel electrophoresis and a UV spectrophotometer. First-stand cDNAs were synthesized using the PrimeScript™ RT reagent kit (TaKaRa) according to the instructions, and qPCR was performed on the CFX96TM real-time PCR system (Bio-Rad). The *gapdh* gene was selected as the internal control, and all samples were subjected to three technical replicates using the 2^−∆∆CT^ method. Significance test analysis was performed using SPSS19 software.

## Results

### Characteristics of the Growth- and Sex-Related Traits

In this study, growth traits were measured and sexes were identified in all 130 offspring of the map family. The mean values of BW, FL, BL, TL, CW, and CHW were 2.60 ± 2.22 g, 4.60 ± 1.16 cm, 3.90 ± 0.98 cm, 1.70 ± 0.41 cm, 0.95 ± 0.26 cm, and 0.30 ± 0.13 cm, respectively. Pearson’s correlation analysis showed that all traits were highly significantly correlated with each other except chelae width. It indicated that the highest significant correlation value was observed between body length and carapace width (*r* = 0.985), while the correlation values between chela width and all other traits were less than zero (Table 1). The mapping family was revealed to consist of 63 males and 67 females with the sex ratio of 1:1.06. Based on morphometric measurement, it was suggested that the growth rate of the males was greater than that of the females under the same culture conditions (Table 2). All of these phenotype data related with growth traits were used as mapping panels for QTL analysis.

**TABLE 1:** Pearson’s correlation between growth traits in F1 progeny (n = 130).

Traits	BW (gm)	FL (cm)	BL (cm)	TL (cm)	CW (cm)	CHW (cm)
BW	1					
FL	0.935	1				
BL	0.937	0.986	1			
TL	0.893	0.959	0.960	1		
CW	0.941	0.983	0.985	0.953	1	
CHW	−0.040	−0.106	−0.100	−0.095	−0.112	1

**TABLE 2:** Comparison of male and female growth traits.

Sex	Number	BW (g)	FL (cm)	BL (cm)	TL (cm)	CW (cm)	CHW (cm)
Female	67	2.23 ± 1.73	4.46 ± 0.64	3.74 ± 0.49	1.64 ± 0.40	0.91 ± 0.25	0.28 ± 0.11
Male	63	2.97 ± 2.62	4.81 ± 1.20	4.03 ± 1.02	1.74 ± 0.44	0.99 ± 0.28	0.32 ± 0.15

### 2b-RAD Sequencing and SNP Genotyping

A total of 348.6 million reads were used, of which 287.4, 30.4, and 30.8 million were from 130 progenies, the male parent, and the female parent, respectively. All of these data have been submitted to the Sequence Read Archive (SRA) database of the NCBI (NCBI accession number: PRJNA778942). After sequential quality filtering and sequence trimming, two parents’ reads were clustered into 561,392 representative high-quality reference tags. Comparing with the reference sequence, a total of 29,364 SNPs were obtained. These SNPs were divided into three categories: paternal heterozygous (lm×ll, 6,391 SNPs), maternal heterozygous (nn×np, 2,789 SNPs), and both heterozygous (hk×hk, 5,767 SNPs). After filtering the SNPs to remove the segregation distorted markers, the remaining 6,948 loci were used to construct a consensus map.

### High-Density Linkage Map

**FIGURE 1:**
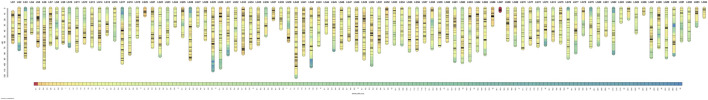
Genetic lengths and marker distributions of 94 linkage groups in the linkage map of red swamp crayfish. The scaleplate on the left indicates genetic distance (centimorgan as the unit). The color module represents the SNP marker distribution density of each centimorgan.

The genetic linkage map, containing 4,878 SNP markers distributed at 4,327 different loci, were successfully divided into 94 LGs, which was consistent with the haploid chromosome number of red swamp crayfish (2n = 188) (Zhang et al., 2018). The consensus map spanned 6,157.737 cM, with an average SNP interval of 1.26 cM. The genetic length of LGs ranged from 5.01 (LG67) to 122.15 (LG40) cM, with an average length of 65.51 cM. The number of markers in each group ranged from 12 (LG83, LG86) to 126 (LG40) with an average of 51.89. The expected map lengths were 6,276.18 cM (*Ge1*) and 6,428.84 cM (*Ge2*), respectively, the average expected map length was 6,352.51 cM (*Ge*), and the genome coverage of this genetic map was 96.93% (Supplementary Table S1; Figure 1).

### QTL Mapping for Growth Traits

**FIGURE 2:**
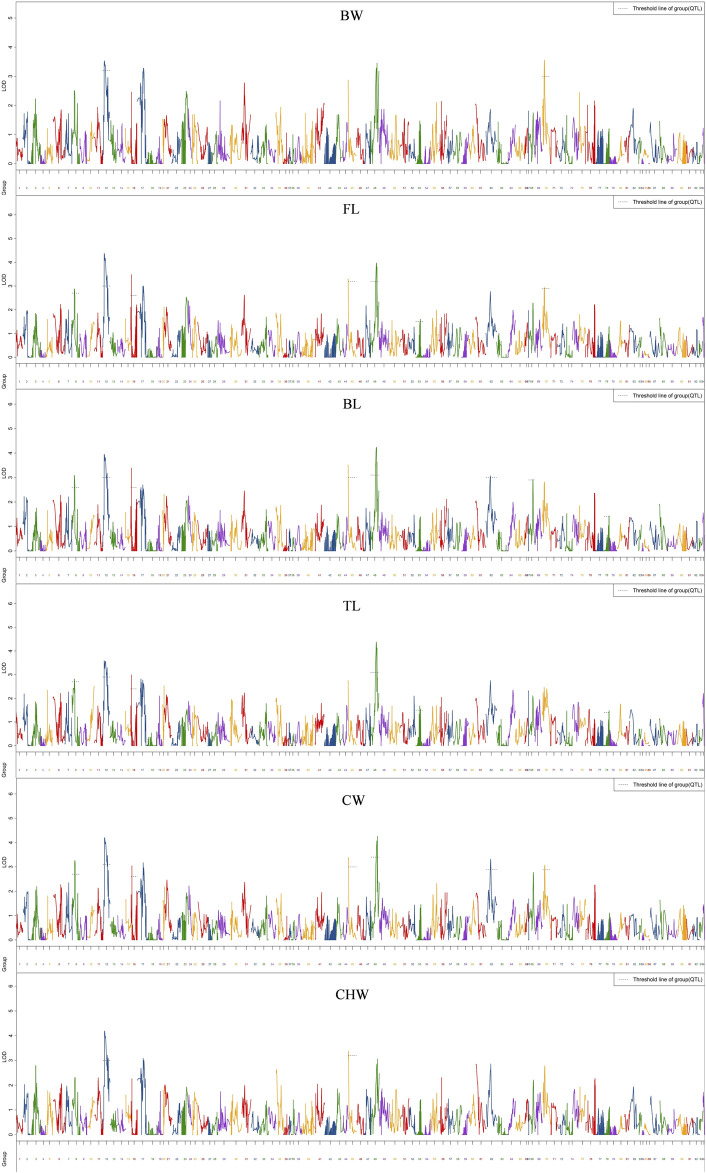
QTL mapping of six growth-related traits of red swamp crayfish. The length of each linkage group is plotted as the coordinate distance in the *x*-axis.

**FIGURE 3:**
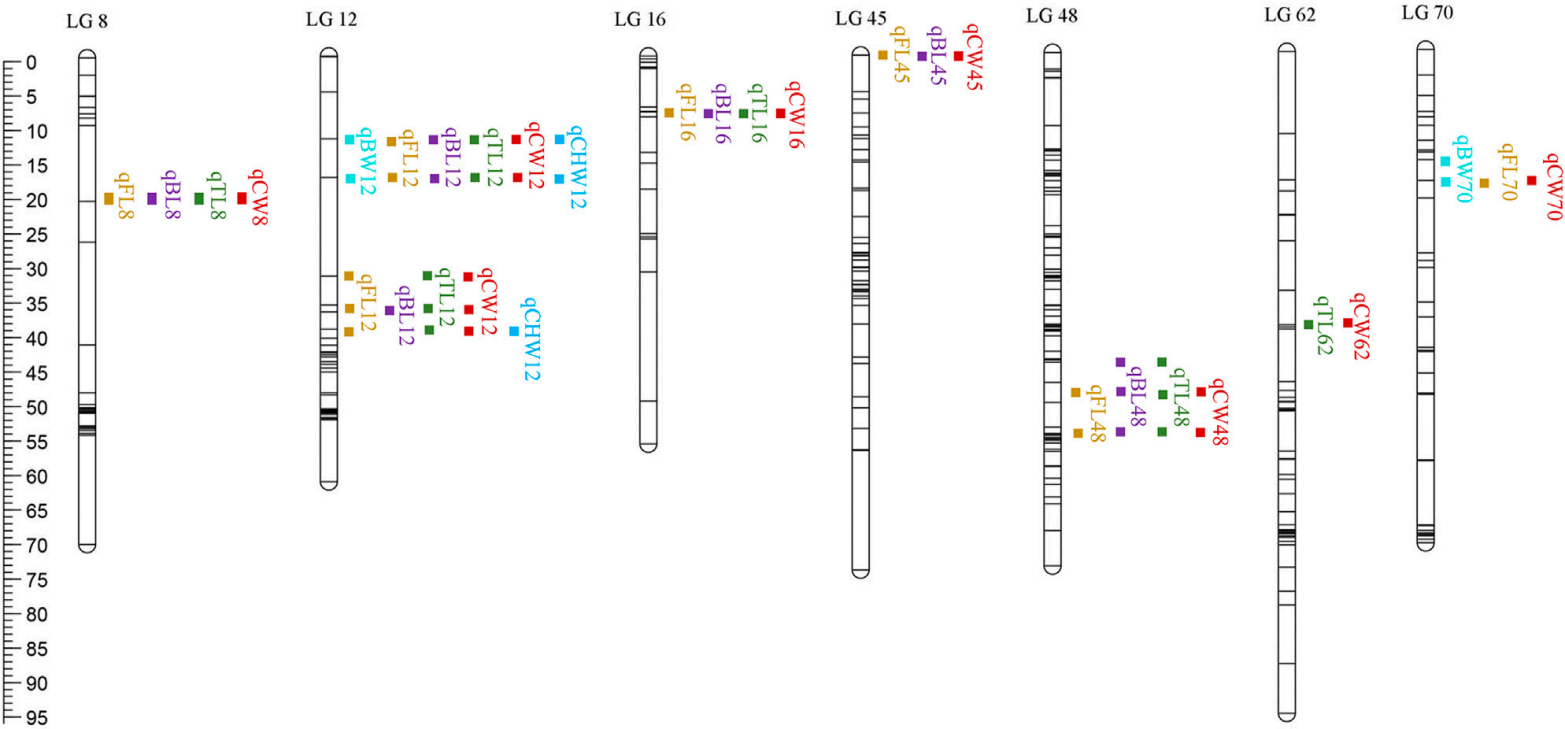
QTL distribution on nine different LGs. The scaleplate on the left indicates genetic distance (centimorgan as the unit).

QTL analysis of six growth-related traits including BW, FL, BL, TL, CW, and CHW of crayfish was performed using MapQTL6 software. QTL fine mapping based on the abovementioned consensus map showed that a total of 28 group-wide significant QTLs associated with growth traits were identified in nine LGs (LG8, 12, 16, 45, 48, 62, 67, 68, and 70), with LOD scores ranging from 2.32 to 4.38, and a phenotypic variance explained (PVE) ranging from 7.9 to 14.4% (Table 3; Figure 2). Each QTL interval harbors 1–2 SNP markers. In detail, two QTLs associated with BW including five SNPs were identified on two LGs (LG12 and LG70), with LOD scores ranging from 3.26 to 3.56 and contributed values of phenotypic variance explained (PVE) ranging from 10.9 to 11.8%. For FL, six QTLs including 14 SNPs were identified on six LGs (LG8, LG12, LG16, LG45, LG48, and LG70), and the most significant QTL located at LG12 with the highest LOD value of 4.36 explained 14.4% of PVE. A total of seven QTLs associated with BL were detected on seven LGs (LG8, LG12, LG16, LG45, LG48, LG62, and LG68) containing 19 SNPs with LOD scores ranging from 2.91 to 4.21 and PVE ranging from 9.9 to 14%. For TL, 19 SNPs from five LGs (LG8, LG12, LG16, LG48, and LG67) were identified with LOD scores ranging from 2.32 to 4.38 and PVE ranging from 7.9 to 14.4%. For CW, a total of 19 SNPs from seven LGs (LG8, LG12, LG16, LG45, LG48, LG62, and LG70) were identified, and the highest LOD score was located in LG12 with highest 14% PVE. Four SNPs from two CHW-related QTLs were identified on LG12, and the most important QTL region was located at 11.98–39.603 cM (LOD = 4.17) explaining 14% of the PVE. All the QTLs also indicated that many overlapping regions were identified (Figure 3). Notably, LG12 was associated with all growth traits, which suggested that these traits may be regulated by genes on LG12, and LG12 was strongly associated with growth. In addition, a total of 11 genes were detected by searching against the genome of red swamp crayfish using extended SNP markers sequences, such as anti-lipopolysaccharide factor 4 (*alf4*), laminin receptor (*lamr*), golgin subfamily B member 1-like (*golgb1*), TBC1 domain family member 1-like (*tbcd1l*), glutamate receptor subunit 1-like (*glurl*), molt-inhibiting hormone (*mih*), and hyperglycemic peptide 2 precursor (*cprp2*) (Table 3).

**TABLE 3:** Summary information of growth-related QTL and candidate genes.

Trait	LG	QTL	Position (cM)	No. of SNPs	Max LOD	LOD threshold		Max PVE%	Candidate gene name
						Group wide	Genome wide		
BW	12	qBW12	11.980–12.037	2	3.47	3.2	6.9	11.6	Anti-lipopolysaccharide factor 4 (*alf4*)
			17.604	1	3.35	3.2	6.9	11.2	\
	70	qBW70	16.003–18.998	2	3.56	3	6.9	11.8	\
FL	8	qFL8	20.78	1	2.88	2.7	5.3	9.8	\
	12	qFL12	11.980–12.037	2	4.36	3	5.3	14.4	Anti-lipopolysaccharide factor 4 (*alf4*)
			17.604	1	4.06	3	5.3	13.5	\
			31.85	1	3.25	3	5.3	11	\
			37.115–39.603	2	3.33	3	5.3	11.2	\
	16	qFL16	7.995	1	3.48	2.6	5.3	11.7	Laminin receptor (*lamr*)
	45	qFL45	0	1	3.25	3.2	5.3	11	\
	48	qFL48	47.83	1	3.47	3.2	5.3	11.7	Golgin subfamily B member 1-like (*golgb1*)
			50.706	1	3.91	3.2	5.3	13	TBC1 domain family member 1-like (*tbcd1l*)
			54.258	1	3.44	3.2	5.3	11.5	Glutamate (NMDA) receptor subunit 1-like (*glurl*)
			57.525	1	3.8	3.2	5.3	12.7	Molt-inhibiting hormone (*mih*)
	70	qFL70	18.998	1	2.9	2.9	5.3	9.8	\
BL	8	qBL8	20.78	1	3.09	2.6	5.3	10.4	\
	12	qBL12	11.980–12.037	2	3.94	3	5.3	13.1	Anti-lipopolysaccharide factor 4 (*alf4*)
			17.604	1	3.67	3	5.3	12.3	\
			37.115	1	3.06	3	5.3	10.3	\
	16	qBL16	7.995	1	3.39	2.6	5.3	11.4	Laminin receptor (*lamr*)
	45	qBL45	0	1	3.5	3	5.3	11.8	\
	48	qBL48	43.314–44.424	2	3.53	3.1	5.3	11.8	Hyperglycemic peptide 2 precursor (cprp2)
			44.601–44.902	2	3.5	3.1	5.3	11.8	Xpa xeroderma pigmentosum, complementation group A (*xpa*)
			47.83	1	3.94	3.1	5.3	13.1	Golgin subfamily B member 1-like (*golgb1*)
			50.706	1	4.21	3.1	5.3	14	TBC1 domain family member 1-like (*tbcd1l*)
			54.258	1	3.69	3.1	5.3	12.4	Glutamate (NMDA) receptor subunit 1-like (*glurl*)
			57.525	1	3.64	3.1	5.3	12.2	Molt-inhibiting hormone (*mih*)
	62	qBL62	39.552	1	3.07	3	5.3	10.4	Nucleosome-remodeling factor subunit NURF301-like (*nurf301*)
			39.934–40.193	2	3.03	3	5.3	10.3	RNA-directed DNA polymerase from mobile element jockey-like
	68	qBL68	38.682	1	2.91	2.9	5.3	9.9	\
TL	8	qTL8	20.78	1	2.82	2.7	5.3	9.5	\
	12	qTL12	11.980–12.037	2	3.59	2.9	5.3	11.9	Anti-lipopolysaccharide factor 4 (*alf4*)
			17.604	1	3.26	2.9	5.3	10.9	\
			31.85	1	2.99	2.9	5.3	10.1	\
			37.115–39.603	2	3.04	2.9	5.3	10.2	\
	16	qTL16	7.995	1	2.99	2.4	5.3	10.1	Laminin receptor (*lamr*)
	48	qTL48	43.314–44.424	2	3.51	3.1	5.3	11.7	Hyperglycemic peptide 2 precursor (cprp2)
			44.601–44.902	2	3.5	3.1	5.3	11.7	Xpa xeroderma pigmentosum, complementation group A (*xpa*)
			47.83	1	3.87	3.1	5.3	12.8	Golgin subfamily B member 1-like (*golgb1*)
			50.706	1	4.38	3.1	5.3	14.4	TBC1 domain family member 1-like (*tbcd1l*)
			54.258	1	3.78	3.1	5.3	12.5	Glutamate (NMDA) receptor subunit 1-like (*glurl*)
			56.585	1	3.39	3.1	5.3	11.3	Enoyl-CoA hydratase (*ech*)
			57.525–57.806	2	4.12	3.1	5.3	13.6	Molt-inhibiting hormone (*mih*)
	67	qTL67	3.473	1	2.32	2.3	5.3	7.9	\
CW	8	qCW8	20.78	1	3.17	2.7	5.3	10.6	\
	12	qCW12	11.980–12.037	2	4.19	3.1	5.3	13.8	Anti-lipopolysaccharide factor 4 (*alf4*)
			17.604	1	3.94	3.1	5.3	13	\
			31.85	1	3.2	3.1	5.3	10.7	\
			37.115–39.603	2	3.42	3.1	5.3	11.4	\
	16	qCW16	7.995	1	3.04	2.6	5.3	10.2	Laminin receptor (*lamr*)
	45	qCW45	0	1	3.31	3	5.3	11	\
	48	qCW48	47.83	1	3.55	3.4	5.3	11.8	Golgin subfamily B member 1-like (*golgb1*)
			50.706	1	3.93	3.4	5.3	13	TBC1 domain family member 1-like (*tbcd1l*)
			54.258	1	3.74	3.4	5.3	12.4	Glutamate (NMDA) receptor subunit 1-like (*glurl*)
			57.525	1	4.26	3.4	5.3	14	Molt-inhibiting hormone (*mih*)
	62	qCW62	39.552	1	3.32	2.9	5.3	11.1	Nucleosome-remodeling factor subunit NURF301-like (*nurf301*)
			39.934–40.193	2	3.25	2.9	5.3	10.9	RNA-directed DNA polymerase from mobile element jockey-like
	70	qCW70	18.998	1	3.06	2.9	5.3	10.3	\
CHW	12	qCHW12	11.980–12.037	2	4.17	3	6	14	Anti-lipopolysaccharide factor 4 (*alf4*)
			17.604	2	3.91	3	6	13.2	
			39.603	1	3	3	6	10.3	

### QTL Mapping for Sex and Screening of Candidate Sex Dimorphic Genes

**FIGURE 4:**
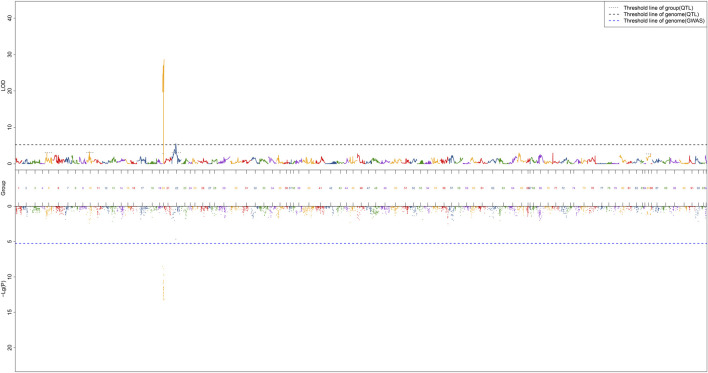
QTL mapping of sex traits of red swamp crayfish. The length of each linkage group is plotted as the coordinate distance in the *x*-axis.

QTL fine mapping revealed one genome-wide QTL interval in LG20 associated with sex containing a total of 31 SNPs, with LOD scores ranging from 19.71 to 28.60 and PVE ranging from 50.3 to 63.7%. The sex-related QTL interval ranged from 0 to 12.9 cM, and the most significant QTL region was located on LG20 at 12.841–12.9 cM with the highest LOD score of 28.6, explaining 63.7% of the PVE (Figure 4). In particular, 28 of 31 SNP markers were female heterozygotes (genotype nn×np), which suggested that crayfish might have a female heterogametic ZW sex determination system.

**FIGURE 5:**
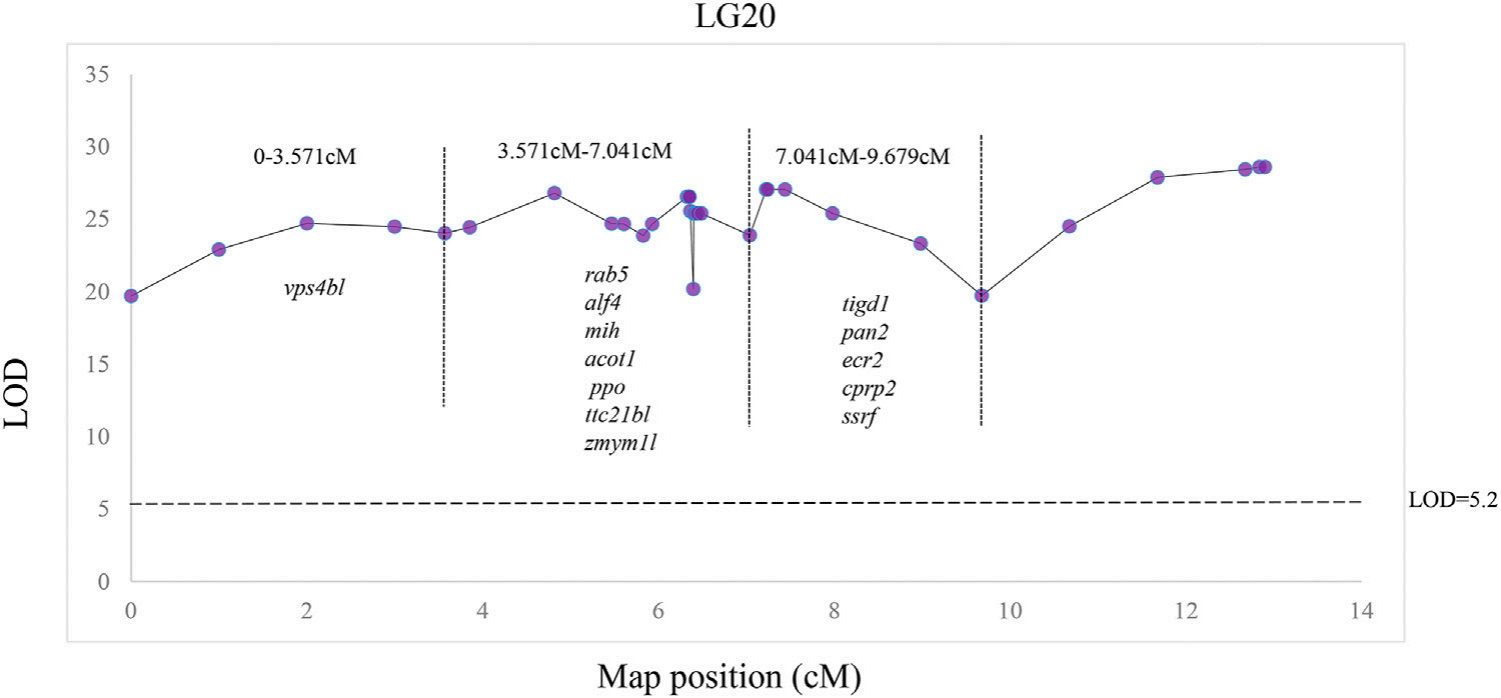
Distribution of 2b-RAD-seq SNP markers and candidate sex dimorphic genes in LG20.

A total of 13 potential candidate genes were further identified from sex-related QTL intervals by searching against the genome of red swamp crayfish using extended SNP markers sequences (Table 4; Figure 5), such as vacuolar protein sorting-associated protein 4B-like (*vps4bl*), acyl-coenzyme A thioesterase 1 (*acot1*), anti-lipopolysaccharide factor 4 (*alf4*), molt-inhibiting hormone (*mih*), tetratricopeptide repeat protein 21B-like (*ttc21bl*), and tigger transposable element-derived protein 1-like (*tigd1*). It is worth noting that *mih* and *alf4* are also growth-related genes from QTL mapping for growth traits, suggesting a possible correlation between growth and gender.

**TABLE 4:** Summary information of sex-related QTL and candidate genes.

LG	QTL	Position (cM)	No. of SNPs	Max LOD	LOD threshold		Max PVE%	Candidate gene name
					Group wide	Genome wide		
20	qSex20	0–3.571	2	24.02	2.8	5.2	57.3	Vacuolar protein sorting-associated protein 4B-like (*vps4bl*)
		3.855–5.609	4	26.81	2.8	5.2	61.3	Small GTPase Rab5 (*rab5*)
		5.827–6.328	3	26.55	2.8	5.2	61	Anti-lipopolysaccharide factor 4 (*alf4*)
		6.344–6.350	3	26.55	2.8	5.2	61	Molt-inhibiting hormone (*mih*)
		6.353–6.398	4	26.55	2.8	5.2	61	Acyl-coenzyme A thioesterase 1 (*acot1*)
		6.402	1	25.4	2.8	5.2	59.3	Prophenoloxidase (*ppo*)
		6.403–6.429	4	25.4	2.8	5.2	59.3	Tetratricopeptide repeat protein 21B-like (*ttc21bl*)
		6.449	1	25.4	2.8	5.2	59.3	Anti-lipopolysaccharide factor 4 (*alf4*)
		6.49–7.041	2	25.4	2.8	5.2	59.3	Zinc finger MYM-type protein 1-like (*zmym1l*)
		7.227	1	27.05	2.8	5.2	61.6	Tigger transposable element-derived protein 1-like (*tigd1*)
		7.24	1	27.05	2.8	5.2	61.6	PAB-dependent poly(A)-specific ribonuclease subunit PAN2 (*pan2*)
		7.44	1	27.05	2.8	5.2	61.6	Ecdysone receptor 2 (*ecr2*)
		7.983	1	25.4	2.8	5.2	59.3	Hyperglycemic peptide 2 precursor (*cprp2*)
		9.679	1	19.71	2.8	5.2	50.3	Spermatogonial stem-cell renewal factor (*ssrf*)
		12.841–12.9	2	28.6	2.8	5.2	63.7	\

### Validation of Candidate Genes by qRT- PCR Analysis

**FIGURE 6:**
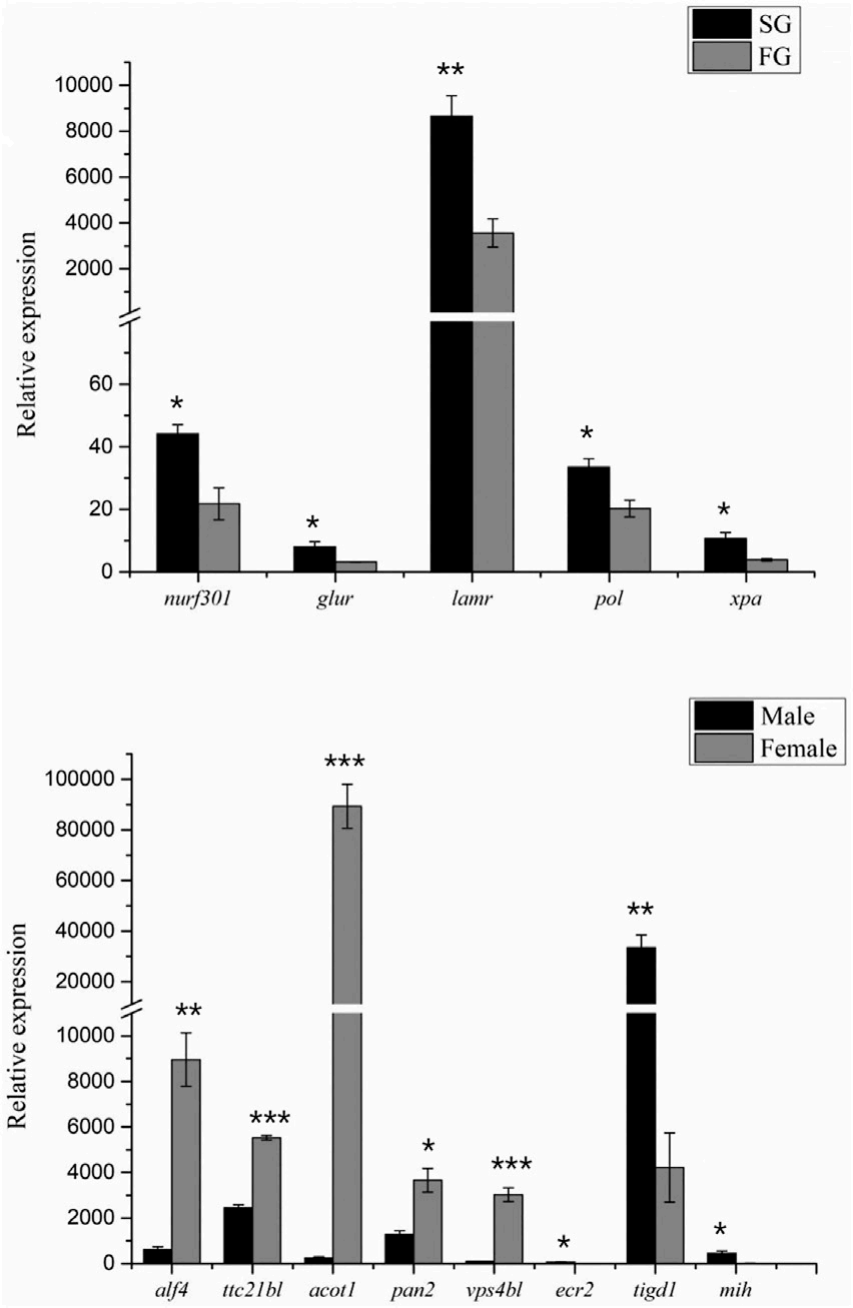
qPCR validation of growth and sex candidate genes. “***,” significant at *p* < 0.001. “**,” significant at *p* < 0.01. “*,” significant at *p* < 0.05.

Based on the abovementioned QTL mapping, a total of 11 growth-related and 13 sex-related potential genes were obtained. The obtained genes were subjected to qPCR and showed that six growth-related genes were significantly differentially expressed in crayfish of different sizes, and eight sex-related genes were differentially expressed in male and female crayfish (Figure 6).

## Discussion

A high-density linkage map is an important and effective tool for QTL fine mapping, comparative genomic analysis, and genome assembly. Compared with former common molecular markers including SSR and AFLP, SNP markers are the most abundant type of genetic markers in the genome with high genetic stability, which are an ideal choice for constructing genetic linkage maps (Berthier-Schaad et al., 2007; Bourgeois et al., 2013). However, it is a great challenge to develop a sufficient number of SNP markers in a relatively large mapping population. 2b-RAD-seq, one of next-generation sequencing (NGS) technologies, is based on type IIB restriction endonuclease enzymatic cleavage of the genome and substantially reduces the complexity of the target genome, especially for the non-model organisms without a reference genome (Wang et al., 2012). To date, high-density linkage maps constructed based on 2b-Rad have been applied in many aquatic animals, such as red-tail catfish (Zhou et al., 2021) and snakehead (Liu et al., 2020). In this study, we constructed the first genetic linkage map of red swamp crayfish by the 2b-RAD-seq technique. The genetic linkage map contained 4,878 SNP markers with an overall genetic length of 6,157.737 cM and an average marker interval of 1.26 cM, as well as 96.93% genome coverage, which could be considered as a high-density genetic map. To date, this is the first report on genetic linkage map of red swamp crayfish. Furthermore, all the SNP markers were assigned to 94 linkage groups, which indicated that the constructed genetic linkage map of red swamp crayfish contained the largest number of linkage groups compared with the previously reported genetic map.

Growth is one of the important economic traits and priority traits for genetic improvement (Bjarne, 1986; Gui and Zhu, 2012). Traditional genetic improvement strategies for growth traits mainly rely on phenotypic and individual selection, which has a long cycle time and low selection efficiency (Tong and Sun, 2015). Meanwhile, molecular marker-assisted breeding uses the characteristics of molecular markers closely linked to the genes determining the target traits to achieve the purpose of selecting the target traits by detecting molecular markers that can save time and cost and improve the selection efficiency (Collard and Mackill, 2008). QTL mapping based on a high-density linkage map could identify potential candidate genes related with the growth traits in many fish species, which provides an effective approach for breeding programs (Tong and Sun, 2015). In this study, we identified 28 group-wide significant QTLs associated with six growth traits in nine LGs. We also found that most of the QTL loci were overlapped and mainly located in several important linkage groups, which may result from the high phenotypic correlation between these traits of crayfish. It also suggested that these traits may be controlled by some common genes. The same observations were revealed in other species, such as red-tail catfish (Zhou et al., 2021), *Hypophthalmichthys nobilis* (Fu et al., 2016), and *Pungitius pungitius* L. (Laine et al., 2013). In addition, each of the six growth traits had 2–7 QTLs with low PVE values of 7.9–14%, suggesting that the growth phenotypic variations of crayfish may be regulated by many QTLs and multiple genes.

A total of 11 candidate growth-related genes from 28 growth-related QTL regions were identified d by QTL mapping. It has been reported that TBC1 domain family member 1 (*tbcd1*) is involved in the regulation of glucose transporter protein 4 transport and glucose uptake in adipocytes and skeletal muscle (Treebak et al., 2010), and downregulation of *tbcd1* is associated with enhanced glucose metabolism in mouse skeletal muscle (Okazaki et al., 2020). Golgin subfamily B member 1 (*golgb1*) encodes golgi-associated large transmembrane protein, and it suggests that a novel 65bp was significantly associated with chicken body weight and significantly associated with neck weight and abdominal fat weight (Linstedt and Hauri, 1993; Fu et al., 2020). Enoyl-CoA hydratase (*ech*) catalyzes the second step of an important physiological oxidative pathway in fatty acid metabolism, and it is involved in growth and development (Agnihotri and Liu, 2003). Crustacean growth is discontinuous and greatly influenced by the molting cycle, and it has been revealed that *mih* mainly regulates the molting process by inhibiting the synthesis of ecdysteroid (Jung et al., 2013). Moreover, SNPs in *mih* were found to be significantly associated with growth traits in both white shrimp and red swamp crayfish (Li et al., 2011; Xu et al., 2019). Moreover, *nurf301* is required for ecdysteroid signaling and metamorphosis, and it is suggested that it is a direct effector of nuclear receptor activity (Badenhorst et al., 2005; Nakatsuji et al., 2009). In addition, qPCR results showed that *nurf301*, *glur*, *lamr*, *pol, xpa*, and *alf4* were expressed differently in FG and SG groups. Therefore, we hypothesized that these genes also regulate growth and development in crayfish.

Many aquatic animals display significant growth differences between males and females; therefore, mono-sex population culture is an effective way to improve economic value (Mei and Gui, 2015; Gui et al., 2022). Sex determination is an important research field in developmental biology and evolutionary biology, and it is a plastic developmental process (Charlesworth and Mank, 2010; Li and Gui, 2018). Sex determination in crustaceans shows degrees of plasticity, being influenced both genetic and other epigenetic factors including environmental variables such as light and temperature, parasites, and even diet (Ford, 2008). Although the sex determination system of some crustaceans was revealed by karyotype analysis (Lécher et al., 1995), it is too difficult to distinguish sex chromosomes by karyotype analysis of the sex determination mechanism due to super genome complexity such as small size, large number, and highly condensed chromosomes in red swamp crayfish (Torrecilla et al., 2017). Therefore, sex-related QTL mapping and sex-specific marker identification are potential methods for sex-associated gene identification and sex determination mechanism uncovering. Sex determination systems of some crustaceans have been revealed by QTL mapping, such as the XX/XY sex determination system in swimming crab (*Portunus trituberculatus*) (Lv et al., 2018) and the ZZ/ZW sex determination system in black tiger shrimp (*Penaeus monodon*) (Guo et al., 2019). In this study, one genome-wide significant QTL related to sex was successfully detected in LG20, explaining 50.3–63.7% of the phenotypic variation, which suggested that sex determination is a single-point trait and employs a specific sex chromosome in red swamp crayfish. In particular, 28 of 31 identified markers were female heterozygotes (genotype nn×np), strongly suggesting that red swamp crayfish may have the female heterogametic ZZ/ZW sex determination system.

Sex determination genes are conducive to uncover the sex characteristics in aquatic animals; therefore, some sex determination genes have been identified (Bao et al., 2019; Trant et al., 2001; Matsuda et al., 2002; Yokoi et al., 2002; Zhou et al., 2002). However, no sex determination genes have been identified in red swamp crayfish to date. In this study, a total of 13 potential genes were detected within the sex QTL region, for example, *ppo*, *vps4bl, acot1, rab5, ttc21bl*, *and ssrf* (Table 4). Prophenoloxidase encoded by *ppo* was detected to be different between the females and the males in *Callosobruchus maculatus*is, but it was suggested that prophenoloxidase was an important defense for invertebrates. We also identified a spermatogonial stem-cell renewal factor (*ssrf*), which could induce spermatogonial mitosis to maintain long-term fertility and sustain sufficiently high levels of spermatogenesis (Mäkelä and Hobbs, 2019). Interestingly, *mih* and *alf4* were identified in both growth-related and sex-related QTLs, which suggested an inevitable correlation existed between growth and sex. Although these sexually dimorphic genes expressed differentially between the males and the females by qPCR validation (Figure 6), they have not been revealed to be necessary for sex determination in other aquatic animals. So, we predicted that the sex determination gene in red swamp crayfish might be other novel genes and it needed more comprehensive and systematic research in the future.

## Conclusion

In this study, 4878 SNP markers distributed in 94 LGs were developed to construct a consensus linkage map, which represents the largest linkage map to date. Abundant QTLs related to growth and sex were identified, uncovering the sexual growth dimorphism and sex determination mechanism. Moreover, our findings will provide effective markers for MAS in red swamp crayfish in the future.

## Data Availability

The datasets presented in this study can be found in online repositories. The names of the repository/repositories and accession number(s) can be found at: https://www.ncbi.nlm.nih.gov/, PRJNA778942.
