# A high-density genetic map and QTL fine mapping for growth- and sex-related traits in red swamp crayfish (*Procambarus clarkii*)

**DOI:** 10.3389/fgene.2022.1113119

**Published:** 2023-01-04

**Authors:** Xin-Fen Guo, Yu-Lin Zhou, Min Liu, Zhi Li, Li Zhou, Zhong-Wei Wang, Jian-Fang Gui

**Affiliations:** ^1^ State Key Laboratory of Freshwater Ecology and Biotechnology, Hubei Hongshan Laboratory, The Innovation Academy of Seed Design, Institute of Hydrobiology, Chinese Academy of Sciences, Wuhan, China; ^2^ College of Life Sciences, University of Chinese Academy of Sciences, Beijing, China; ^3^ Key Laboratory of Ministry of Water Resources for Ecological Impacts of Hydraulic Projects and Restoration of Aquatic Ecosystem, Institute of Hydroecology, Ministry of Water Resources, Chinese Academy of Sciences, Wuhan, China

**Keywords:** *Procambarus clarkii*, genetic linkage map, QTL mapping, growth-related traits, sex

Incorrect Funding

In the published article, there was an error in the **Funding** statement. [This work was supported by grants from the National Key R and D Program of China (2018YFD0901201) and Key R&D projects in Hubei Province (2021BBA232)]. The correct **Funding** statement appears below.

Funding

[This work was supported by grants from the National Key R and D Program of China (2018YFD0901303) and Key R&D projects in Hubei Province (2021BBA232)].

The authors apologize for this error and state that this does not change the scientific conclusions of the article in any way. The original article has been updated.

