# Supplementary material for: A High-Density Genetic Map and QTL Fine Mapping for Growth- and Sex-Related Traits in Red Swamp Crayfish (Procambarus clarkii)
Source: Front Genet. 2022 Feb 15;13:852280. doi: 10.3389/fgene.2022.852280 (PMC8886229; doi:10.3389/fgene.2022.852280)
Supplement: Supplementary file 1 [file Table1.DOCX]

Supplementary Table S1 Detail information of genetic linkage map

| Linkage group | Mapped markers | Distinct positions | Genetic length(cM) | Marker interval(cM) |
| --- | --- | --- | --- | --- |
| LG1 | 77 | 69 | 60.366 | 0.78 |
| LG2 | 67 | 57 | 73.113 | 1.09 |
| LG3 | 94 | 84 | 86.781 | 0.92 |
| LG4 | 18 | 17 | 44.118 | 2.45 |
| LG5 | 108 | 93 | 66.808 | 0.62 |
| LG6 | 99 | 90 | 106.447 | 1.08 |
| LG7 | 67 | 60 | 64.109 | 0.96 |
| LG8 | 31 | 29 | 70.515 | 2.27 |
| LG9 | 53 | 45 | 61.672 | 1.16 |
| LG10 | 41 | 36 | 67.569 | 1.65 |
| LG11 | 49 | 45 | 76.338 | 1.56 |
| LG12 | 41 | 36 | 61.652 | 1.5 |
| LG13 | 53 | 48 | 68.045 | 1.28 |
| LG14 | 69 | 63 | 72.679 | 1.05 |
| LG15 | 36 | 32 | 46.525 | 1.29 |
| LG16 | 25 | 22 | 56.176 | 2.25 |
| LG17 | 82 | 77 | 97.148 | 1.18 |
| LG18 | 41 | 35 | 81.495 | 1.99 |
| LG19 | 40 | 35 | 51.187 | 1.28 |
| LG20 | 45 | 39 | 12.9 | 0.29 |
| LG21 | 59 | 54 | 68.943 | 1.17 |
| LG22 | 57 | 52 | 84.738 | 1.49 |
| LG23 | 27 | 25 | 63.203 | 2.34 |
| LG24 | 19 | 17 | 32.036 | 1.69 |
| LG25 | 47 | 37 | 50.223 | 1.07 |
| LG26 | 78 | 73 | 90.848 | 1.16 |
| LG27 | 23 | 18 | 31.372 | 1.36 |
| LG28 | 43 | 37 | 55.413 | 1.29 |
| LG29 | 88 | 79 | 110.535 | 1.26 |
| LG30 | 75 | 68 | 105.612 | 1.41 |
| LG31 | 60 | 48 | 82.073 | 1.37 |
| LG32 | 45 | 43 | 74.299 | 1.65 |
| LG33 | 118 | 99 | 80.609 | 0.68 |
| LG34 | 51 | 47 | 70.66 | 1.39 |
| LG35 | 68 | 61 | 64.343 | 0.95 |
| LG36 | 32 | 25 | 48.619 | 1.52 |
| LG37 | 34 | 31 | 19.946 | 0.59 |
| LG38 | 25 | 21 | 36.175 | 1.45 |
| LG39 | 54 | 51 | 58.176 | 1.08 |
| LG40 | 126 | 110 | 122.15 | 0.97 |
| LG41 | 98 | 91 | 82.572 | 0.84 |
| LG42 | 56 | 48 | 103.322 | 1.85 |
| LG43 | 46 | 42 | 65.753 | 1.43 |
| LG44 | 26 | 22 | 43.808 | 1.68 |
| LG45 | 48 | 45 | 74.604 | 1.55 |
| LG46 | 59 | 49 | 71.49 | 1.21 |
| LG47 | 40 | 37 | 56.656 | 1.42 |
| LG48 | 86 | 75 | 74.405 | 0.87 |
| LG49 | 101 | 92 | 89.779 | 0.89 |
| LG50 | 86 | 76 | 100.772 | 1.17 |
| LG51 | 74 | 64 | 77.882 | 1.05 |
| LG52 | 74 | 63 | 61.96 | 0.84 |
| LG53 | 32 | 28 | 63.545 | 1.99 |
| LG54 | 33 | 28 | 64.618 | 1.96 |
| LG55 | 61 | 57 | 75.846 | 1.24 |
| LG56 | 76 | 69 | 74.001 | 0.97 |
| LG57 | 60 | 52 | 59.546 | 0.99 |
| LG58 | 47 | 42 | 71.539 | 1.52 |
| LG59 | 50 | 44 | 66.535 | 1.33 |
| LG60 | 59 | 57 | 58.646 | 0.99 |
| LG61 | 67 | 61 | 93.868 | 1.4 |
| LG62 | 58 | 52 | 95.937 | 1.65 |
| LG63 | 64 | 56 | 93.051 | 1.45 |
| LG64 | 68 | 62 | 75.438 | 1.11 |
| LG65 | 61 | 52 | 81.902 | 1.34 |
| LG66 | 60 | 52 | 30.218 | 0.5 |
| LG67 | 60 | 40 | 5.011 | 0.08 |
| LG68 | 59 | 53 | 56.679 | 0.96 |
| LG69 | 51 | 48 | 65.871 | 1.29 |
| LG70 | 41 | 38 | 71.519 | 1.74 |
| LG71 | 41 | 36 | 63.64 | 1.55 |
| LG72 | 53 | 46 | 67.495 | 1.27 |
| LG73 | 43 | 39 | 65.035 | 1.51 |
| LG74 | 39 | 36 | 57.062 | 1.46 |
| LG75 | 46 | 42 | 61.153 | 1.33 |
| LG76 | 39 | 33 | 88.228 | 2.26 |
| LG77 | 40 | 40 | 78.779 | 1.97 |
| LG78 | 36 | 32 | 46.841 | 1.3 |
| LG79 | 33 | 27 | 68.33 | 2.07 |
| LG80 | 32 | 30 | 64.95 | 2.03 |
| LG81 | 24 | 21 | 48.237 | 2.01 |
| LG82 | 25 | 24 | 80.441 | 3.22 |
| LG83 | 12 | 11 | 24.501 | 2.04 |
| LG84 | 23 | 22 | 18.764 | 0.82 |
| LG85 | 17 | 16 | 43.37 | 2.55 |
| LG86 | 12 | 10 | 10.423 | 0.87 |
| LG87 | 58 | 49 | 86.934 | 1.5 |
| LG88 | 55 | 50 | 69.499 | 1.26 |
| LG89 | 50 | 40 | 87.479 | 1.75 |
| LG90 | 47 | 42 | 82.831 | 1.76 |
| LG91 | 20 | 19 | 57.635 | 2.88 |
| LG92 | 24 | 22 | 56.604 | 2.36 |
| LG93 | 28 | 24 | 31.558 | 1.13 |
| LG94 | 15 | 13 | 15.559 | 1.04 |
| Total | 4878 | 4327 | 6157.737 | 1.26 |
| *G*e | 6352.51 |  |  |  |
| coverage | 96.93% |  |  |  |
